# Economic evaluation of continuous subcutaneous insulin infusion for children with diabetes - a pilot study: CSII application for children – economic evaluation

**DOI:** 10.1186/1471-2431-13-155

**Published:** 2013-10-01

**Authors:** Elina Petkova, Valentina Petkova, Maia Konstantinova, Guenka Petrova

**Affiliations:** 1 Department of Social Pharmacy, Medical University Sofia, Faculty of Pharmacy, Sofia, Bulgaria; 2 Medical University Sofia, University Pediatric Hospital, Endocrinology Clinic, Sofia, Bulgaria

**Keywords:** Pediatric diabetes, Continuous subcutaneous insulin infusion (CSII), Insulin analogues, Cost-effectiveness analysis

## Abstract

**Background:**

The objective of this study is to assess the cost of using continuous subcutaneous insulin infusion to treat children with type-1diabetes in Bulgaria, considering changes in body mass index (BMI) and the glycated hemoglobin. The study was performed from the perspective of the Bulgarian National Health Insurance Fund (NHIF) and patients.

**Methods:**

A total of 34 pediatric type-1-diabetes patients were observed for 7 months, divided into 2 groups – on pumps and on insulin analogue therapy. Patient demographic data, BMI and glycated hemoglobin level were obtained and recorded. The cost of insulin, pumps, and consumables were calculated and compared with changes in glycated hemoglobin level. The incremental cost-effectiveness ratio was below the threshold value of gross domestic product per capita.

**Results:**

The results were sensitive to changes in glycated hemoglobin level. Improvements associated with glycemic control led to a reduced glycated hemoglobin level that could ensure good diabetes management, but its influence on BMI in growing children remains unclear.

**Conclusion:**

Continuous subcutaneous insulin infusion appears to be more cost-effective for the Bulgarian pediatric population and health care system.

## Background

Type-1-diabetes (T1DM) patients treated with unmodified regular human insulin (RHI) rarely achieve their glycemic target and often suffer from postprandial hyperglycemic incidents, together with an increased risk of hypoglycemia in the post-absorptive period [1]. Recent meta-analyses in the literature have found improved glycemic control with continuous subcutaneous insulin infusion (CSII) compared with multiple daily injections (MDI) of insulin for patients with diabetes mellitus. For example, in Australia, CSII is predominantly used in type-1-diabetes mellitus patient populations [2]. Continuous subcutaneous insulin infusion (CSII) is considered an option for type-1diabetic patients unsatisfactorily controlled with multiple daily injections (MDI). Short-acting analogs are superior to regular human insulin in CSII. There is evidence supporting the advantages of short-acting analog-based CSII over MDI in type-1 diabetes. The reduction of glycated hemoglobin (HbA1c) level with CSII was evident in trials enrolling patients with mean age greater than 10 years [3].

The main goals for managing children with type-1-diabetes mellitus include achieving near-normal blood sugar levels, minimizing hypoglycemic incidents, optimizing quality of life, and preventing or delaying long-term complications. Continuous subcutaneous insulin infusion (CSII) is a treatment option that can assist in achieving all of these goals in all ages of children [4]. European Union countries reimburse insulin therapy for individuals with health insurance, but for CSII reimbursement, a variety of approaches exist [5-7]. The objective of this study is to assess the cost of using CSII of insulin to treat children with type-1diabetes in Bulgaria and to compare it with the changes in BMI and HbA1c. The study was performed from the perspective of the Bulgarian NHIF and patients. The main study question discussed is "will the use of CSII be cost-effective for the Bulgarian health care system?"

## Methods

### Literature search

PubMed was searched using keywords CSII, type-1diabetes, pediatric population and all articles analyzing the safety, efficacy, and cost-effectiveness of CSII usage in the pediatric population were selected. In total, 4 studies were selected and their objectives, methodologies, results and conclusions were compared [2,8-10].

### Type of the CSII usage study

A combined retrospective and prospective analysis of children patient records after the introduction of CSII was performed based on the patients’ records and observation. This study was performed at the Endocrinology Clinic of University pediatric hospital of the Medical University, Sofia. It was reviewed and approved by the Ethics committee of the Science medical council of the Medical University in Sofia.

### Patient selection

A total of 34 children with type-1diabetes were observed divided into two groups: with an active group using CSII and a control group using analogue insulin therapy with a pen device. Thirty children in the country use CSII, and of these, 17 were surveyed, after their parents signed informed-consent forms. The children were consecutively recruited from the end of 2007 when the first pumps were administered. The active group included all children who began using the CSII pumps during the period 2007–2011 when the data collection began. Also since 2010, all children were transferred to real time insulin pumps; therefore at the moment of observation, they all used the same type of pump from the same manufacturer.

The control group was formed after reviewing patient records and random selection according to age, duration of diabetes, entrance BMI and HbA1c level. Their parents also signed informed-consent forms.

### Data collection

The data was collected by observing the therapeutic effects on both groups from the Endocrinology pediatric clinic from 01.02.2012 to 31.08.2012 (7 months). During this period, we measured the diabetes maintenance phase after CSII introduction. Data for the selected children was collected on their demographics, age, gender, weight, duration of disease, therapeutic schema (CSII or analogue insulin treatment with a pen device) and HbA1c before the inclusion in the pump program, and at the end of the observation.

### Cost – effectiveness analysis

For both groups of children, the health care resources used by them were recorded, namely insulin, pumps (1 for 4 years), consumables for pumps (6–10 sets and 6–10 reservoirs), strips (n = 1100 per patient per year), glucometers (1 for 5 years including sensor prices), GP and endocrinology visits, and hospitalization due to diabetes. Sensors were used from 7 to 10 days. Yearly costs of CSII, blood glucose monitoring systems, insulin therapy, and strips were calculated by multiplying the number of resources used by their prices. Prices of pumps and blood glucose monitoring systems were collected from the manufacturers’ websites. To calculate the yearly pump costs, the prices were divided by 4, which is recommended by the manufacturers as the period of use for initial users [11]. All other costs were taken from the Bulgarian NHIF tariff [12]. Costs are presented in Bulgarian leva (BGN). At the time, the exchange rate was 1 Euro: 1.95 BGN.

The primary outcome observed was the change in HbA(1c) before the pump introduction and at the end of the study. The secondary outcome observed was the BMI change during the same period. In this pilot study we did not include the hypoglycemia episodes due to lack of data for all children. Children were introduced to pumps in different time periods, and in order to calculate the corresponding decrease in the HbA1c level, the total decrease during the period was divided into the duration of the period when the particular child was using the pump. Finally the average decrease for both groups was calculated.

Cost effectiveness ratio (CER) was calculated by dividing the yearly cost of the health care resources and the changes in the HbA1c level. Incremental cost-effectiveness ratio (ICER) was also calculated by dividing the differences in costs between the active and control group with the differences in the HbA1c level.

### Sensitivity analysis

To test the robustness of the results, a one-way sensitivity analysis was performed by consecutively varying the changes in the HbA1c within the standard deviation interval for both groups of patients with 0.05.

### Statistical analysis

Descriptive statistics were applied to the patient’s characteristic and outcomes. A *T*-test analysis was also performed to test the statistical significance in the outcome changes.

## Results

### Analysis of published studies

There are 4 studies in the literature that discuss the efficacy, safety and/or cost-effectiveness of CSII usage in the pediatric population – Table 1. The number of observed patients varied from 19 to 95. All of these studies measured the decrease in HbA1C, and some, in addition, focused on patients’ demographic characteristics, glucose level, hypoglycemia, and quality of life. All of the studies conclude that CSII is safe and effective, leading to greater decreases in HbA1c levels, allowing for improved quality of life, decreased hypoglycemic events and improved child and parent adherence as shown in Table 1.

**Table 1:** Summary of the main findings from the published studies

**Authors**	**Objective**	**Methodology**	**Results**	**Conclusions**
Plotnick et al. [8]	To evaluate the safety and effectiveness of insulin pump therapy in children and adolescents with type-1diabetes.	All patients who started insulin pump therapy between 1 January 1990 and 31 December 2000 were included in this study. Medical records were reviewed for 95 patients, ages 4–18 years at pump start. The mean (SD) age was 12.0-3.1 years, and children under the age of 10 years comprised 29% of the group. Patients and families chose insulin pump therapy for several reasons, including better control, less blood glucose variability, fewer injections and improvement in lifestyle flexibility. HbA1c was measured at each visit by cation-exchange high-performance liquid chromatography.	There was a small but significant decrease in HbA1c at 3–6 months after starting with pump (7.7 vs. 7.5%; *P* < 0.03). HbA1c levels then gradually increased and remained elevated after 1 year of follow- up. This association was confounded by age and diabetes duration, both of which were associated with higher HbA1c levels. After adjusting for duration and age, mean HbA1c after pump start was significantly lower than before pump start (7.7 vs. 8.1%; *P* <0.001). There were fewer hypoglycemic events after pump start (12 vs. 17, rate ratio 0.46, 95% CI 0.21–1.01).	Insulin pump use was safe and effective.
				After adjusting for age and duration of diabetes, HbA1c was in fact lower after pump placement.
				Both monitoring frequency and parental involvement were significantly associated with lower HbA1c levels.
Bode et. al. [9]	To compare multiple daily injections (MDI), and CSII and to assess the effects on quality of life.	Comparative analysis	In adults and adolescents with type-1diabetes, CSII has been shown to lower HbA1c levels, reduce the frequency of severe hypoglycemia and limit excessive weight gain versus MDI without increasing the risk of diabetic ketoacidosis. The effectiveness of CSII and improvements in pump technology have fueled a dramatic increase in the use of this therapy.	Insulin pump or continuous subcutaneous insulin infusion (CSII) therapy provides a treatment option that can dramatically aid in achieving all of these goals.
Wilson et al. [10]	To compare continuous subcutaneous insulin infusion (CSII), and continuing multiple daily injections (MDIs), in respect to their safety in young children, glycemic control, hypoglycemia and quality of life.	A randomized 1-year feasibility trial comparing CSII with continuing MDIs in preschool children with a history of type-1diabetes for at least 6 months’ duration. Prospective outcomes included measures of overall glycemic control (HbA1c and continuous glucose monitoring system), the incidence of severe hypoglycemia and diabetic ketoacidosis, the percent of glucose values below 3.9 mmol/l, and the parents’ report of quality of life.	The 19 subjects’ ages ranged from 1.7 to 6.1 (mean 3.6) years, duration of diabetes ranged from 0.6 to 2.6 (mean 1.4) years, and baseline HbA1c ranged from 6.7 to 9.6% (mean 7.9%). Nine subjects were randomized to start CSII and 10 to continue on MDI. Overall metabolic control, diabetes quality of life, and the incidence of hypoglycemia were similar in the two groups. No subject had diabetic ketoacidosis, while one subject in each group had an episode of severe hypoglycemia. No CSII subject discontinued using the pump during or after the study.	CSII can be a safe and effective method to deliver insulin in young children.
Cohen et al. [2]	To project long-term costs and outcomes of CSII compared with MDI in adult and adolescent T1DM.	The study modelled analysis utilizing a lifetime horizon in adult and adolescent specialty-care type-1-diabetes patient populations from Australia. Published diabetes complication costs, treatment costs and discount rates of 5.0% per annum were applied to costs and clinical outcomes. A lifetime horizon was used, considering only direct medical costs and excluding indirect and non-medical costs. The validated CORE diabetes model employs standard Markov/Monte Carlo simulation techniques.	Mean direct lifetime outcomes were $A 34 642 higher with CSII treatment than with MDI for adult patients and $A 41 779 for adolescent patients. Treatment with CSII is associated with an improvement in life expectancy of 0.393 years for adults compared with MDI and 0.537 years for adolescents. The corresponding gains in QALYs were 0.467 QALYs and 0.560 QALYs for adults and adolescents, respectively. This produced incremental cost effectiveness ratios (ICERs) of $A88 220 and $A 77851 per life-year gained for CSII compared with MDI for adult and adolescent T1DM.	The analysis suggests that CSII is associated with ICERs in the range of $A53 022–259 646 per QALY gained with most ICERs representing a significant savings in Australia under the majority of scenarios explored.

The published studies define the HbA1c level as the widely accepted measure of diabetes control in pediatric practice. Positive therapeutic results after CSII introduction that might lead to better long-term outcomes are observed.

### Results of the national study

The University pediatric clinic has been introducing CSII on the request of the parents, with only 30 children having applied so far. From 1999 to 2011, 17 children with diabetes type-1 were observed mean age 113,82 months in the active group and 112.,41 in the control group. The duration of diabetes was a little lower in the control group – Table 2.

**Table 2:** Patient demographic

	**Gender**	**Age (months)**	**Months with diabetes**
Insulin	Males (n = 10)	112.41 ± 42.705	41.71 ± 22.79
	Females (n = 7)		
Pumps	Males (n = 9)	113.82 ± 49.054	66.65 ± 41.07
	Females (n = 8)		

In both groups, BMI increased and the change was higher in the active group with lower standard deviation (SD), meaning that these children maintained stable growth. A stable and significant decrease in the HbA1c level is observed in the group of patients using CSII (1.25 ± 0.99). It is also evident that the CSII groups maintained close to target levels of HbA1c (6,5%) at the end of the study, while in the control group, the target control was not observed – Table 3.

**Table 3:** Changes in the study outcomes

	**BMI before**	**BMI after**	**BMI difference**	**HbA1c (%) before**	**HbA1c (%) after**	**HbA1c (%) difference**
Insulin	18 ± 2.716	19.47 ± 2.125	1,47 ± 2,71	10.11 ± 1.46	9.01 ± 2.50	0.52 ± 0.41
Pumps	17 ± 2.739	19.65 ± 1.272	2,65 ± 1,53	8.99 ± 0.66	7,1 ± 0,67	1.25 ± 0.99

The CSII price of the blood glucose monitoring system was 7850 BGN (4025,64 Euro) thus reaching 1962,50 BGN (1006,41 Euro) per patient per year – Table 4. The transmitter cost was 425 BGN (217,95 Euro). The test strips cost 1039,35 BGN (533 Euro) year (1100 strips per year) and their average cost according to the duration of the disease was 7369,93 BGN (3779.45 Euro) from onset and diagnosis. This cost was equal in both groups per protocol and was not included in the cost analysis. Insulin usage due to the strict control was lower in the group of children with CSII and therefore their yearly cost of insulin therapy was lower (Table 3). The total cost of therapy in the group of CSII users was higher mainly due to the CSII pumps and related consumables.

**Table 4:** Cost of the therapy (BGN)

	**Insulin cost**	**Pump cost**	**Transmitter cost**	**Consumables**	**Total**
Insulin	700.92 ± 224.304			225	925,92
Pumps	453.96 ± 144	1962,5	425	3360	6201,46

The cost per unit of decrease in HbA1c in the control group was 1780,91 BGN (913.13 Euro) and almost 3.5 times higher in the group using CSII – Table 5. But the ICER showing the additional cost per unit of decrease in HbA1c was lower than the recommended threshold of yearly gross domestic product (GDP) per capita. This means that CSII usage is cost-effective for the Bulgarian health care system.

**Table 5:** Cost –effectiveness analysis (BGN)

	**Total cost**	**Change in HbA1c**	**CER**	**ICER**
Insulin	925,92	0.52 ± 0.41	1780,61	
Pumps	6201,46	1.25 ± 0.99	4961,17	7226,77

**Figure 1:**
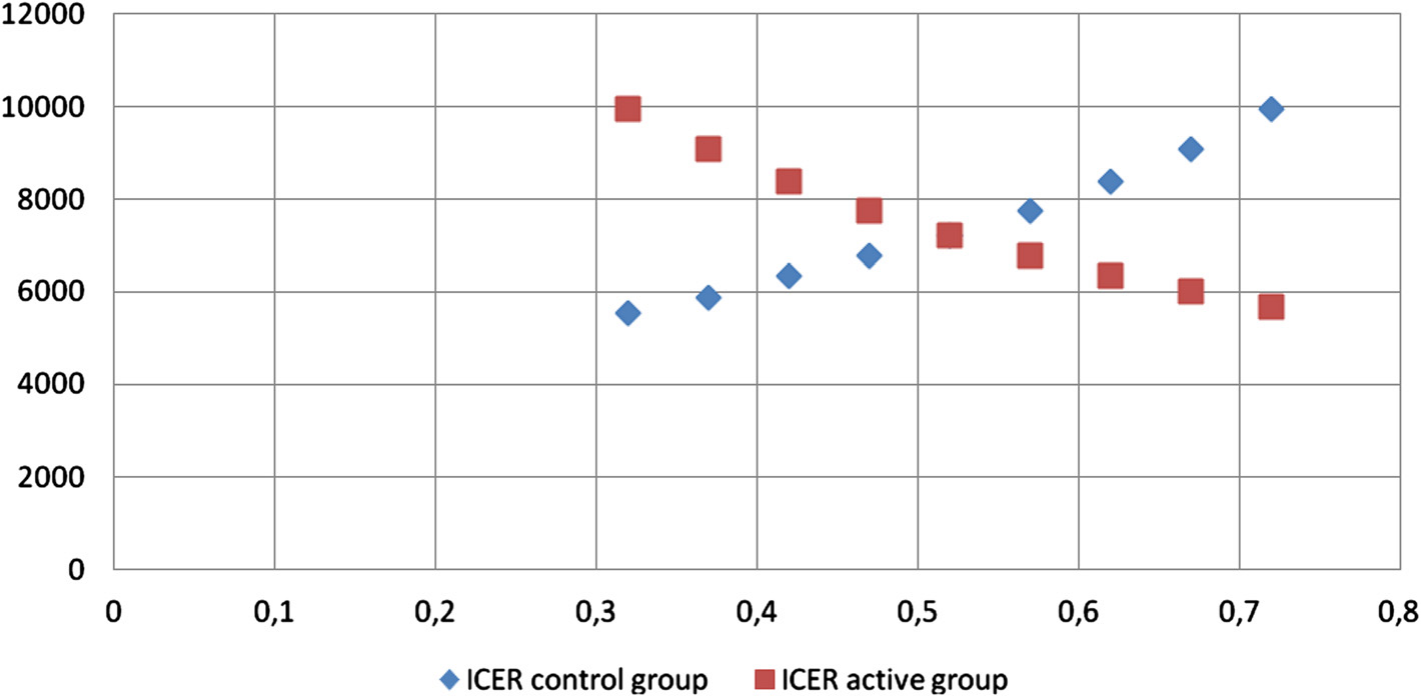
ICER change in active and control group when differences in HbA1c change.

The results were sensitive to changes in the HbA1c level in both groups. The ICER ratio for CSII pumps remained below the threshold value when the difference in HbA1c level was below 0,42% in the control group and 1,30% in the active group. When the differences in the HbA1c level in the control group increased, the ICER also increased, while in the active group, the opposite occurred. This means that the therapy with the pumps is an efficient alternative for the health care system in Bulgaria when children manage to decrease their HbA1c level by more than 1.30% (Figure 1).

## Discussion

Continuous subcutaneous insulin infusion (CSII) systems are of a limited usage because they are not reimbursed by the Health Insurance Fund in Bulgaria. No official criteria for CSII usage in child populations have been established and only parents with sufficiently high income are able to afford such a therapeutic approach. In this sense, evaluation of the cost-effectiveness of CSII usage is influenced by a number of factors, such as health insurance policy, parents’ preferences, and therapeutic standards. Our study shows that the usage of CSII with child populations is an efficient therapy and confirms similar findings reported in the literature on improvements in terms of better metabolic control, reduced rates of complications and better quality of life [2,13].

The study also shows that the children using CSII manage to maintain stable and target HbA1c levels, which are preconditions for better diabetes management (UKPDS, DCCT). The studies of the CSII usage in child populations are very limited for comparing long-term results in detail, but bearing in mind the evidence for the adult population, it can be predicted that strict and reliable disease control for children will support their long-term survival.

One limitation of this study is the small patient sample due to the limited number of children on CSII in Bulgaria, but it includes more than 57% of all the CSII users in the country, which ensures reliable results for the whole group. The number of the patients is twice as high as Wilsons’ study [8]. Some of the costs are not included in the analysis (strips, physicians visits, hospitalizations) because they were equal for the both groups and would not change the ICER. We did not calculate the cost-effectiveness of CSII pumps related to the second observed outcome – BMI, because an increase in children’s weight as a result of their normal developmental growth was observed in the both groups.

Our study aimed to provide evidence for the Bulgarian NHIF to include CSII within the reimbursement system. Strict criteria for appropriate selection of children must be developed, as well as cost controls in order to make the final decision. The reimbursement practice in some countries provides such evidence, as in Serbia, where insurance authorities are paying for consumables and patients’ families are paying for pumps. In countries with high GDP, CSII is included within the scope of the reimbursement system [7].

## Conclusion

Improvements in glycemic control associated with CSII led to reduced HbA(1c), which can ensure good diabetes management, but its control over BMI in growing children remains unclear. CSII pumps appear to be cost-effective for the Bulgarian pediatric population and health care system.

## Competing interests

The authors declare that they have no competing interests.

## Authors’ contributions

EP carried out the data collection of the data. VP participated in the design of the study and performed the document and statistical analysis and helped to draft the manuscript. MK participated in the design and coordination. GP participated in the design of the study and in the statistical analysis and helped to draft the manuscript. All authors read and approved the final manuscript.

## Pre-publication history

The pre-publication history for this paper can be accessed here:

http://www.biomedcentral.com/1471-2431/13/155/prepub
